# Diversity and compositional changes in the gut microbiota of wild and captive vertebrates: a meta-analysis

**DOI:** 10.1038/s41598-021-02015-6

**Published:** 2021-11-22

**Authors:** Antton Alberdi, Garazi Martin Bideguren, Ostaizka Aizpurua

**Affiliations:** Center for Evolutionary Hologenomics, GLOBE Institute, University of Copenhagen, 1353 Copenhagen, Denmark

**Keywords:** Ecology, Evolutionary ecology, Microbial ecology, Microbiology, Bacteria, Microbial communities

## Abstract

The gut microbiota is recognised as an essential asset for the normal functioning of animal biology. When wild animals are moved into captivity, the modified environmental pressures are expected to rewire the gut microbiota, yet whether this transition follows similar patterns across vertebrates is still unresolved due to the absence of systematic multi-species analyses. We performed a meta-analysis of gut microbiota profiles of 322 captive and 322 wild specimens from 24 vertebrate species. Our analyses yielded no overall pattern of diversity and compositional variation between wild and captive vertebrates, but a heterogeneous landscape of responses, which differed depending on the components of diversity considered. Captive populations showed enrichment patterns of human-associated microorganisms, and the minimal host phylogenetic signal suggests that changes between wild and captive populations are mainly driven by case-specific captivity conditions. Finally, we show that microbiota differences between wild and captive populations can impact evolutionary and ecological inferences that rely on hierarchical clustering-based comparative analyses of gut microbial communities across species.

## Introduction

The gastrointestinal tract of most animals on Earth hosts a microbial community^1^, known as the gut microbiota, which shapes their phenotypes through modulating a range of physiological processes^2^. Due to the plasticity of the gut microbiota^3^, animals living in different environmental conditions often exhibit particular microbial signatures^4^. While many wild animals are reared in captivity for reasons spanning scientific research, to education, leisure and conservation, there is growing evidence that conditions in captivity alter the microbial community associated with animals. Some studies reported that the microbial diversity dropped in captivity^5–10^, probably due to the simplification of the environment in which hosts live. However, evidence supporting the opposite pattern has also been reported in some other species^8,11^, although it has been suggested these observations might be spurious^12^. In consequence, whether consistent patterns of gut microbiota variation exist between wild and captive animals remains unresolved.

Besides, captive animals are often used to address ecological and evolutionary questions of animal-microbiota interactions; e.g., how the natural history of vertebrates shape their gut microbiota^13^. Marked differences between wild and captive animals could challenge the representativeness of biological results drawn from captive animals^14^. However, the level of distortion that the use of captive animals entails for such analyses is yet to be assessed.

To address these questions, we analysed the gut microbiota of comparable wild and long-term captive individuals belonging to 24 vertebrate species, including fish, amphibians, reptiles and mammals, and performed a meta-analysis of the diversity and compositional variation between wild and captive individuals. We analysed whether the observed changes across vertebrates exhibited any host phylogenetic signal, and we contrasted the data to five theoretical scenarios relating to how the gut microbiota may vary when comparing wild and captive animals. Aiming at identifying microbial taxa systematically enriched in captive or wild environments, we also performed a meta-analysis of taxonomic relative abundances. Finally, we assessed how ecological and evolutionary inferences can differ depending on the origin of the animal specimens employed to characterise gut microbial communities.

## Results and discussion

**Figure 1:**
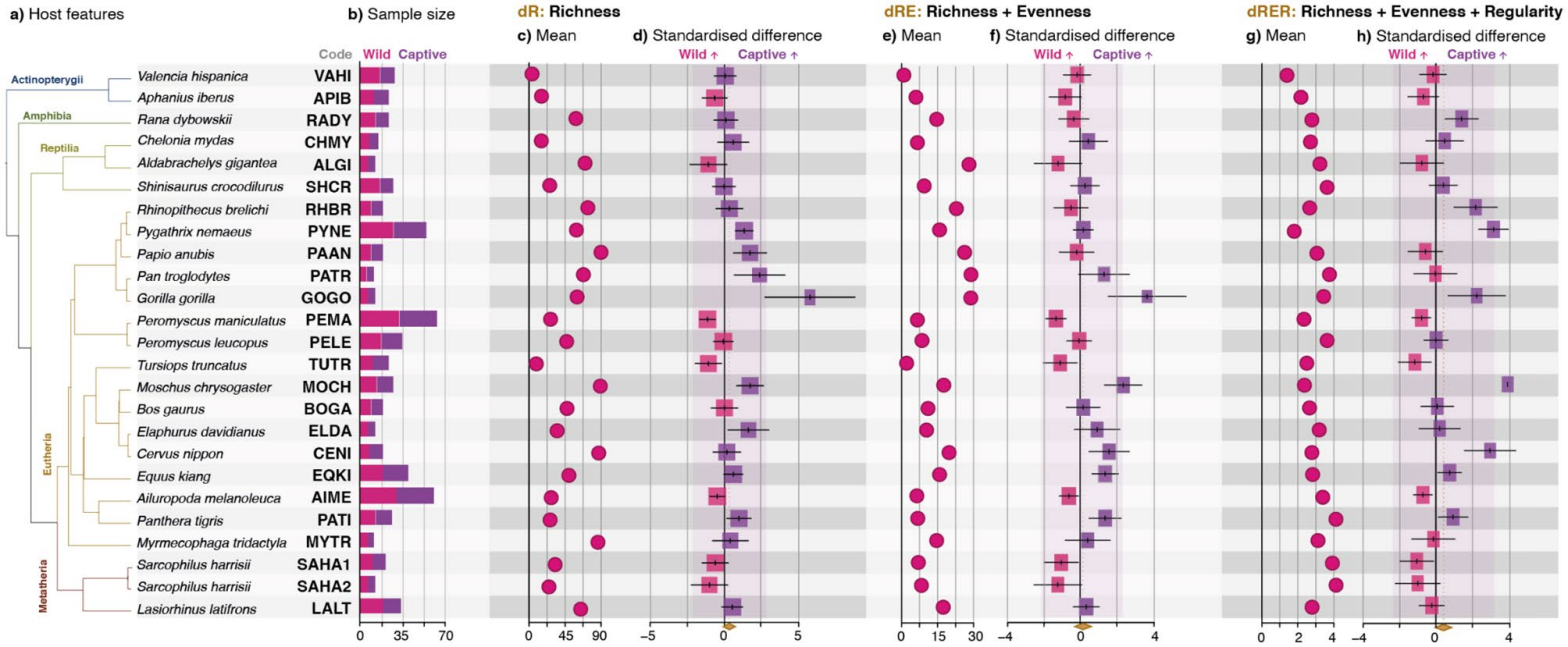
Diversity differences of the gut microbiota between wild and captive vertebrate populations. (**a**) Phylogenetic tree, scientific names and dataset code of the analysed host species. (**b**) Number of wild and captive individuals. (**c**) Mean richness (number of genera) detected. (**d**) Mean standardised difference between the richness (dR) of wild and captive populations. Positive numbers indicate that the captive population is richer than the wild population and vice versa. (**e**) Mean effective number of taxa detected considering richness and eveness components (dRE). (**f**) Mean standardised difference between diversity dRE of wild and captive populations. (**g**) Mean effective number of lineages detected considering richness, eveness and regularity components (dRER). (**h**) Mean standardised difference between phylogenetic diversity dRER of wild and captive populations.

After screening 222 publications, we retrieved raw 16S rRNA gene amplicon data and corresponding metadata from 23 publications that met the criteria explained in the Methods section. To increase comparability^15^, (i) a common bioinformatic pipeline developed to deal with such heterogeneous data was used to re-generate relative abundance information from raw reads, and (ii) sequence data were aggregated to the genus taxonomic level to minimise biases introduced by the distinct 16S rRNA regions targeted^16^. The resulting datasets contained 644 individuals (322 wild and 322 captive; mean of 25.8 individuals/dataset) belonging to 24 vertebrate species (Fig. 1a,b; Supplementary Information).

We conducted diversity analyses based on Hill numbers^17^, using three contrasting metrics that account for different components of diversity, and thus provide insights into the relative contribution of each of these components to the observed changes. dR (Richness) only considers a single component of diversity, namely richness, and thus measures the number of taxa detected. dRE (Richness + Evenness) considers two components of diversity, namely the number of taxa detected and their relative abundances, and measures diversity in effective number of taxa. dRER (Richness + Evenness + Regularity), incorporates a third component, namely regularity, which accounts for the phylogenetic relationships among the bacteria, and thus diversity is measured in effective number of lineages. Further explanations of these metrics can be found in the methods section. For all three studied metrics, alpha (K-W_dR_: X^2^ = 516.715, df = 25, p-value < 0.001; K-W_dRE_: X^2^ = 476.759, df = 25, p-value < 0.001; K-W_dRER_: X^2^ = 337.573, df = 25, p-value < 0.001) and beta (PMV_dR_: R^2^ = 0.509, df = 24, p-value < 0.001; PMV_dRE_: R^2^ = 0.691, df = 24, p-value < 0.001; PMV_dRER_: R^2^ = 0.683, df = 24, p-value < 0.001) diversities of the gut microbial communities differed across host species. The average number of genera (dR) ranged between 3.7 ± 3.2 and 89.4 ± 16.0 (Fig. 1c), while the effective number of genera (dRE) spanned 1.5 ± 0.9 to 28.5 ± 6.5 (Fig. 1e) and the effective number of lineages (dRER) ranged between 1.4 ± 0.7 and 4.1 ± 1.6 (Fig. 1g).

The overall meta-analyses of diversity differences based on the three metrics showed no systematic trend towards neither decreasing nor increasing diversity between wild and captive animals (Random-Effect-Models, REM_dR_: SMD = 0.326, t = 1.370, p-value = 0.184, Fig. 1d; REM_dRE_: SMD = 0.155, t = 0.700, p-value = 0.489, Fig. 1f; REM_dRER_: SMD = 0.414, t = 1.480, p-value = 0.151, Fig. 1h). Neither of the two monophyletic subset of host taxa with the highest representation (5 species) exhibited any systematic trend either (Primates: REM_dR_: SMD = 1.972, t = 2.430, p-value = 0.072; REM_dRE_: SMD = 0.682, t = 1.000, p-value = 0.374; REM_dRER_: SMD = 1.387, t = 1.900, p-value = 0.131; Cetartiodactylans: REM_dR_: SMD = 0.440, t = 0.830, p-value = 0.451; REM_dRE_: SMD = 0.749, t = 1.280, p-value = 0.270; REM_dRER_: SMD = 1.157, t = 1.21, p-value = 0.294). Our results therefore indicate that captivity neither systematically drops, nor increases, the diversity of the gut microbiota, and that increased diversities observed in some species in captivity are not the result of poor study design and limited sample size as previously suggested^12^, but real biological signal. Phylogenetic signal measured by means of Abouheif’s C_mean_ index, showed that closely related host species tended to exhibit more similar responses when measuring richness (p-value = 0.013). This result could indicate that intrinsic features of hosts elicit similar microbiota responses to captivity in related vertebrates, although this pattern could also be the result of employing similar management practices in related animals. In any case, no phylogenetic signal was detected when considering more components of diversity (dRE: p-value = 0.109; dRER: p-value = 0.208), probably because the differences among hosts are less pronounced when more complex diversity metrics are employed (24 × difference between most and least diverse host when measuring dR, and 2.9× difference when measuring dRER). The effect of captivity on the microbial diversity might be influenced by intrinsic features of the host, but it seems to be more heavily impacted by the conditions animals are exposed to, such as the diet, environment and health treatments^18,19^. This level of detail is not always reported in the literature (see Supporting Dataset), which prevented us from assessing the impact extrinsic factors have in microbiota profiles^19^.

**Figure 2:**
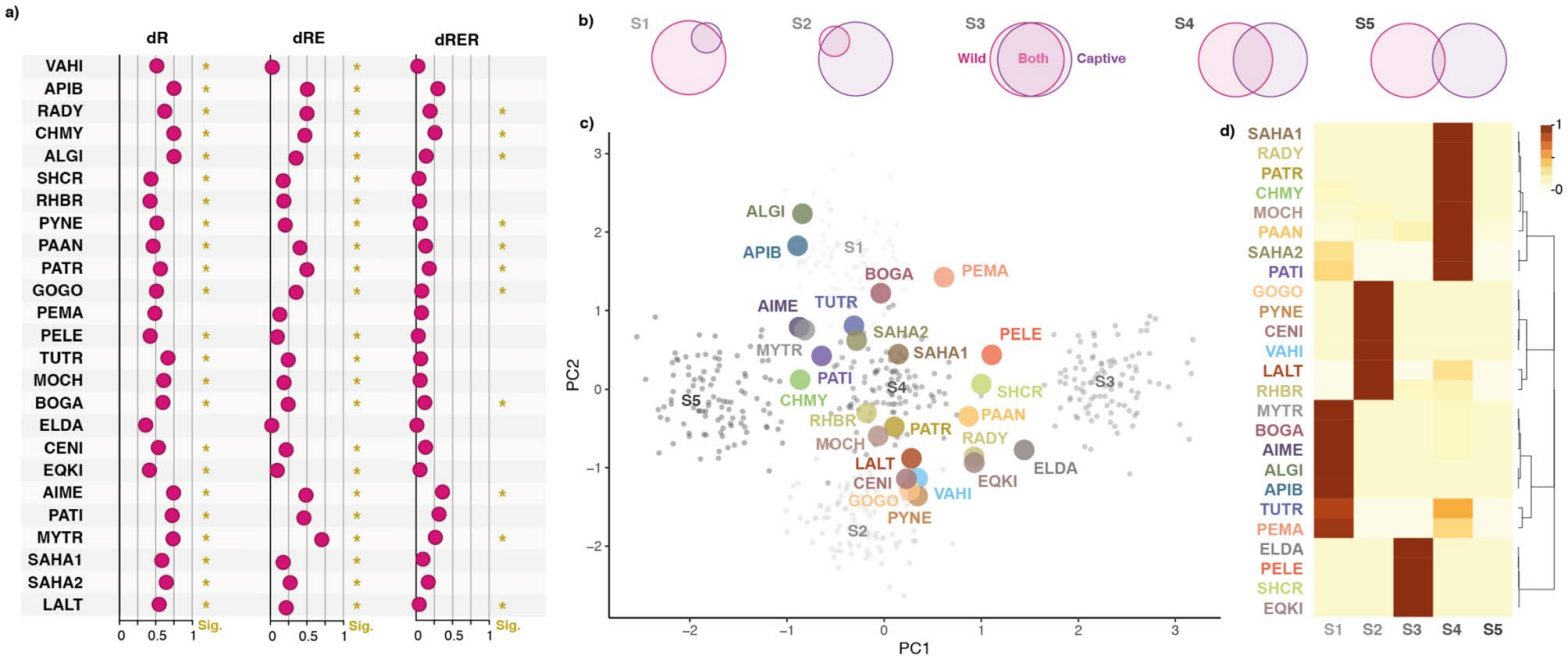
Compositional differences of the gut microbiota between wild and captive vertebrate populations. (**a**) Compositional dissimilarity values between captive and wild populations for the different diversity metrics analysed. Stars indicate whether dissimilarities were significant according to the null models. (**b**) Visual representation of the five scenarios of microbiota variation. “S1” depicts the gut microbiota of captive animals as a subset of that of wild counterparts. “S2” describes the opposite scenario in which the gut microbiota of wild animals is a subset of that of captive counterparts. “S3” assumes barely no difference between the gut microbiota of both populations. “S4” defines a a situation in which captive animals recruit a proportional set of microorganisms that is different from that of wild counterparts, yet maintain a considerable overlap. “S5” describes a scenario in which the gut microbiotas of both populations are almost totally different. (**c**) Principal components analysis showing observed microbiota variation in the studied host species (large coloured dots) over simulated data points (small greyscale dots). (**d**) Histogram of posterior probabilities of the contrasted scenarios for each host species, sorted according to hierarchical clustering dendrogram. Abbreviations are explained in Fig. 1a.

Regarding compositional changes, dissimilarity metrics based on Hill numbers’ beta diversity^17^ exhibited moderate values when analysing richness (dR: 0.57 ± 0.12; 0.34–0.75), but decreased considerably when considering also the evenness (dRE: 0.29 ± 0.18; 0.04–0.70) and regularity (dRER: 0.13 ± 0.10; 0.02–0.38) components (Fig. 2a). This drop suggests that the turnover of genera occurred mainly among bacteria with low relative abundances and with phylogenetically related taxa. Accordingly, in all but two species (*Elaphurus davidianus* and *Peromyscus maniculatu*s), the dissimilarities observed between wild and captive populations were higher than the null expectations (p-index > 0.95) when considering only richness (dR) and richness + evenness (dRE) (Fig. 2a). However, in 11 of the studied species, significance disappeared when considering all three components of diversity (dRER), further supporting that the turnover of bacteria mostly happened among phylogenetically closely related taxa. This suggests that although captivity induces a change of the bacterial community composition, most of the bacteria recruited under captive conditions probably fill the niches left by closely related lost relatives (Fig. 1f,g).

To gain further insights into the structure of compositional changes at the genus level, each dataset was contrasted to five theoretical scenarios (S1-5) of gut microbiota compositional differences between wild and captive animals using approximate Bayesian computation (Fig. 2b). We found that the analysed host species exhibited microbiota variation patterns that resembled four of these scenarios. Eight species (32%) were classified under S4, which defines a situation in which captive animals recruit a proportional set of microorganisms that is different from that of wild counterparts, yet maintain a considerable overlap. The variation observed in seven other species (28%) was classified under S1, which depicts the gut microbiota of captive animals as a subset of that of wild counterparts. The rest of species were classified under S2 (24%) and S3 (16%). Interestingly, none of the species exhibited a (almost) complete turnover of the microbial community, as described in S5. These results show that captivity does not trigger a complete regime shift of the gut microbial community of vertebrates, yet similar to diversity, compositional changes also exhibit a heterogeneous response across vertebrates (Fig. 2c,d). These differences could possibly be the result of the varying degree of complexity to reproduce wild conditions under captivity for different host species. However, the lack of phylogenetic signal in the compositional differences (dR: p-value = 0.163, dRE: p-value = 0.460; dRER: p-value = 0.314) does not support this hypothesis, either because the effects of different captivity management procedures or the variability introduced by different laboratory processing in the analysed studies are larger than intrinsic features of the animals.

**Figure 3:**
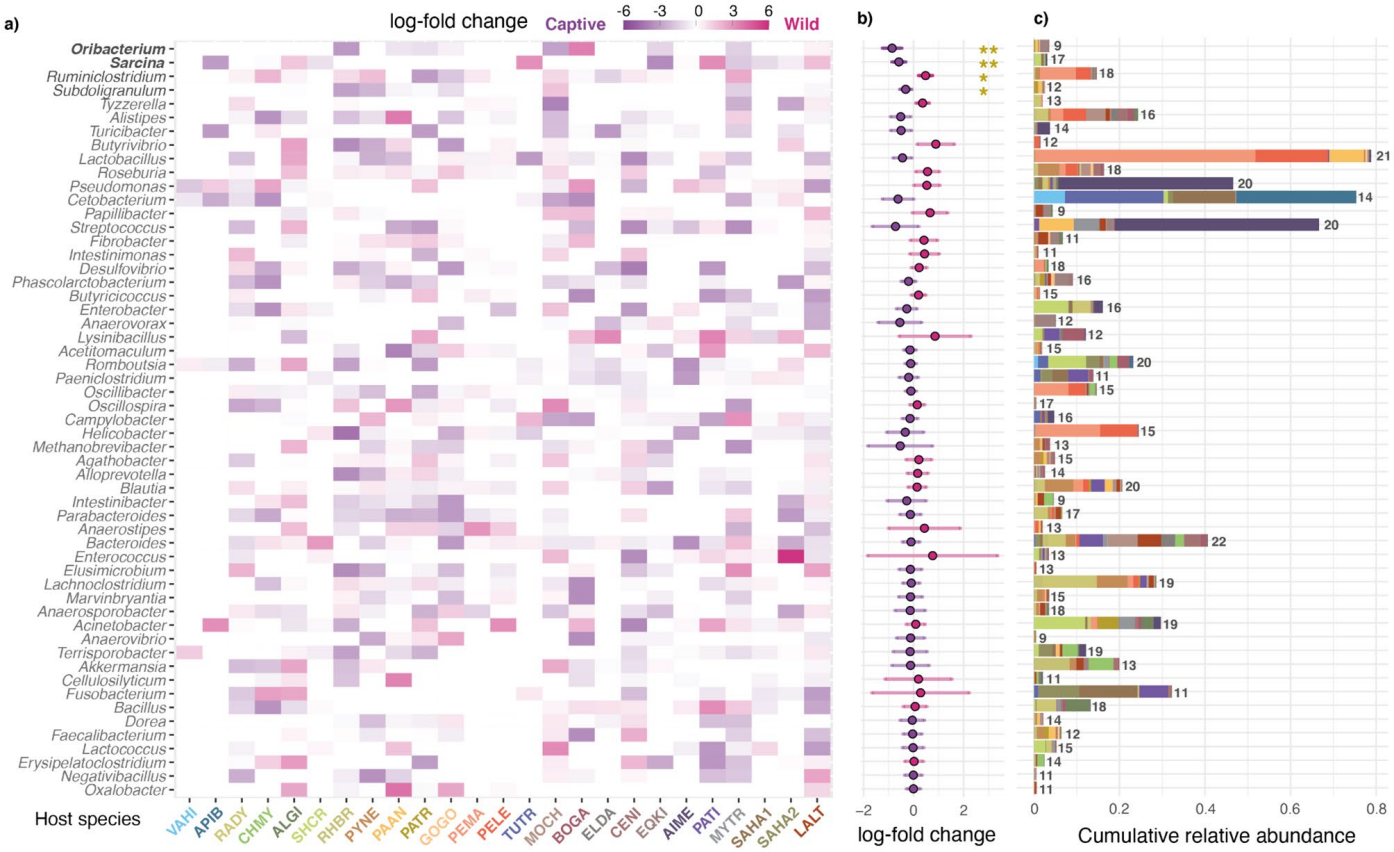
Differential abundance of the microbial taxa with highest prevalence. The listed microbial taxa were detected in more than a 33% (9) of the studies. (**a**) Log-fold changes of the most prevalent taxa in each host species. Purple tones indicate enrichment towards captive conditions, while pink tones indicate enrichment towards wild conditions. (**b**) Log-fold change estimates and their standard errors derived from the random effects meta-analysis of microbial abundances. Two stars indicate p-values under 0.05, while a single star indicate p-values under 0.1. (**c**) Cumulative relative abundances of taxa across the analysed datasets. Each coloured box indicates the mean relative abundance value of the microbial taxa in each study. Numbers indicate number of host species in which each taxon was detected. Abbreviations are explained in Fig. 1a.

We further investigated the taxonomic differences between wild and captive populations across the analysed host species using Generalized Additive Models for Location, Scale and Shape (GAMLSS) models. We identified 55 microbial genera with a host species-level prevalence over 33% (at least present in 9 datasets out of 25) (Fig. 3a). Among these, we observed that more bacteria exhibited enrichment trends towards captivity than wild environments (Fig. 3b). However, statistically significant (p-value < 0.05) or near-significant (p-value < 0.1) log-fold changes were only detected in four bacteria, all belonging to the phylum Firmicutes, and exhibiting somewhat low relative abundances in the gut microbiota of most hosts (Fig. 3c). *Oribacterium*, *Sarcina* and *Subdoligranulum*, which were enriched in captive environments, are common dwellers of the human gastrointestinal tract^20–22^. The same happens with many other taxa that, although beyond significance thresholds, showed enrichment patterns in captive environments (e.g., *Alistipes*, *Turicibacter*, *Lactobacillus*). In contrast, *Ruminiclostridium*, which is a strictly anaerobic taxon that is found in the intestinal tract of ruminants and termites, was the only taxon significantly enriched in wild conditions. All these observations suggest that contact with humans, or exposure to a more anthropised environment, might have contributed to increase the prevalence of human-associated taxa in captive animals. These bacteria do not become dominant taxa in captive animals (Fig. 3c), yet they seem to be important drivers of the microbiota turnover between wild and captive populations.

**Figure 4:**
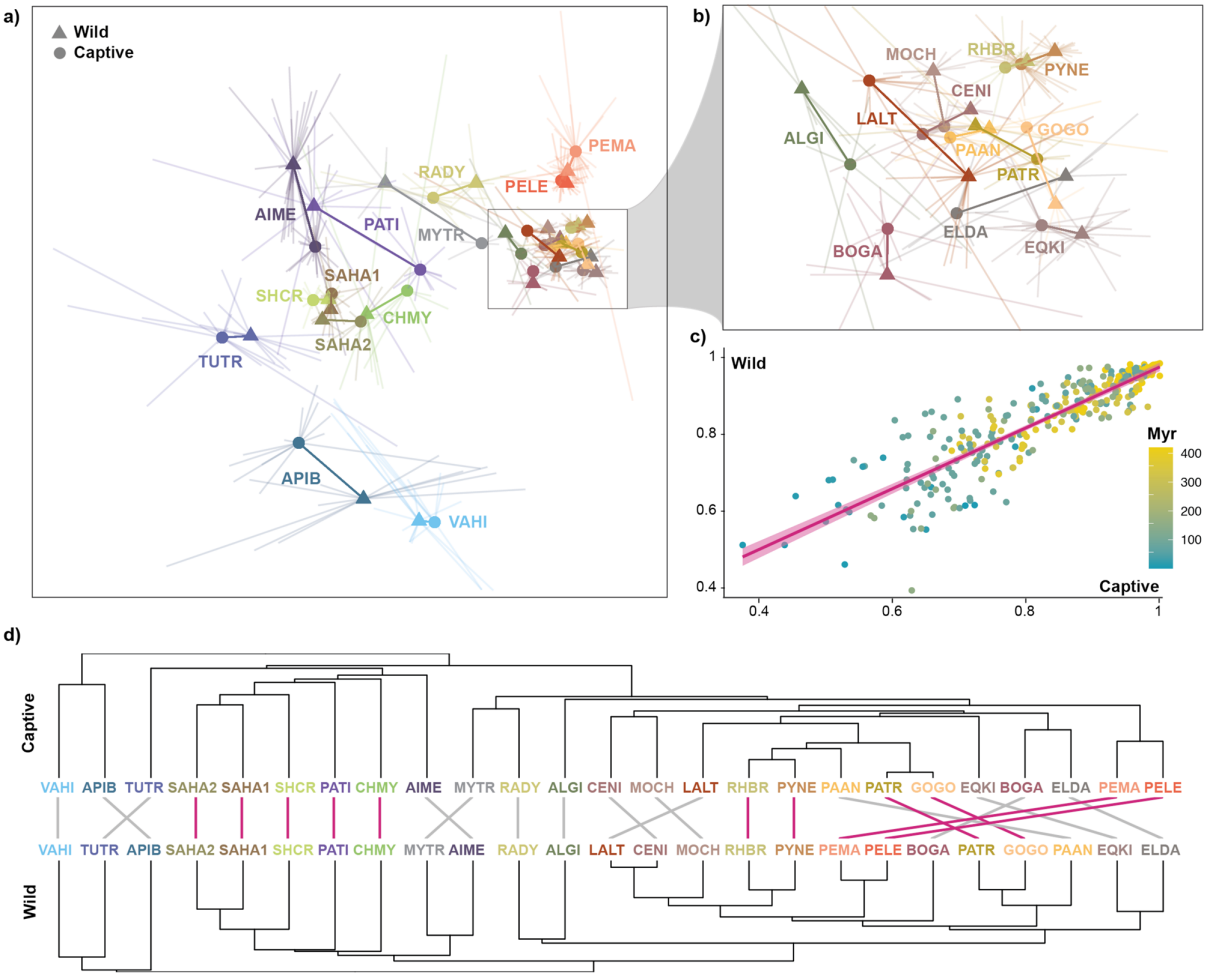
Compositional differences of the gut microbiota between wild and captive animals. (**a**) NMDS plot of the gut microbiota composition between wild and captive animals coloured by host species. Triangles and circles linked by solid lines indicate the centroids of the gut microbiota composition in wild and captive animals, respectively, as defined by the individual data (outer edges of the thin lines) of each species. (**b**) Zoomed image of the NMDS plot to improve the visualisation of compositionally similar primates and artiodactylans. (**c**) Correlation plot between pairwise compositional differences of the gut microbiota between host species as calculated based on wild (Y axis) and captive (X axis) specimens. Colours of the dots indicate the evolutionary distance (in millions of years) between compared hosts. (**d**) Topological differences of the hierarchical clustering of species-level gut microbiota based on captive and wild animals. Common subtrees between captive and wild cladograms are highlighted by pink connecting lines. Abbreviations are explained in Fig. 1a.

Finally, we assessed the impact of such compositional differences in commonly employed procedures used to compare animal-associated microbiotas when addressing ecological and evolutionary questions. Overall, we detected a strong significant correlation between the gut microbiota dissimilarity across species computed based on captive and wild populations (dR: r = 0.877, t = 31.538, df = 298, p-value < 0.001; dRE: r = 0.922, t = 41.097, df = 298, p-value < 0.001; dRER: r = 0.880, t = 31.937, df = 298, p-value < 0.001, Fig. 4c). However, in 30% of the cases, the microbiota of a population from a different species exhibited a more similar composition than the wild or captive counterpart of a particular species (Fig. 4a,b). Our results show that such differences between captive and wild vertebrates yield diverging topologies in hierarchical clustering analyses (Fig. 4d), which result in significantly different phylosymbiosis effect sizes (dR: t = 4.358, df = 193.27, p-value < 0.001; dRE: t = 3.779, df = 194.79, p-value < 0.001; dRER: t = 4.648, df = 195.46, p-value < 0.001), and thus potentially impact inferences on animal-microbiota eco-evolutionary patterns^23^. This is particularly relevant when analysing species with shared dietary features and similar hindgut microbial communities, such as primates and artiodactylans (Fig. 4b).

## Conclusions

Our study revealed that long-term captivity does not induce systematic directional changes of the diversity and composition of the gut microbiota in vertebrates. Instead, host taxa exhibit a heterogeneous landscape of responses, that vary depending on the components of diversity employed to characterise them. This heterogeneity seems to be mainly driven by extrinsic features of captivity conditions, which unfortunately we were unable to associate with microbiota variation due to the lack of metadata on the features and conditions of wild and captive populations. The lack of general patterns suggest that captive populations of different species need to be managed on a case-by-case basis. A relevant aspect to ensure optimal captivity management will be to ascertain whether the observed microbiota changes have a significant physiological impact on their hosts. Faecal microbiota transplants coupled with physiological tests could be used to test this, while shotgun sequencing-based microbiota analyses would contribute to find mechanistic explanations that link microbiota variation with animal health. Another unresolved aspect of the gut microbiota of captive vertebrates is the impact of the human-associated microbial reservoir in the rewiring of their microbial communities. Whether human-associated microbes enriched in captive environments actively oust their wild counterparts, or instead passively fill the niches that lost wild microbes leave, will need to be studied through longitudinal analyses in which temporal dynamics of the gut microbiotas of wild vertebrates moved into captivity are analysed in detail.

Finally, our study also reflects the data and knowledge bias towards mammal-associated microbial communities, which hinders implementation of robust phylogenetic analyses to address evolutionary aspects of the studied topic. More data from non-mammal hosts and an effort to standardise metadata would therefore be desirable to better understand the impact of captivity in the gut microbiota of wild animals. In the meanwhile, as microbiota differences between wild and captive populations can introduce biases in multi-species comparative analyses, data from captive animals should be used with caution when addressing evolutionary questions concerning wild organisms.

## Methods

### Literature search and data retrieval

We performed a systematic literature search on the internet (Google Scholar, Web of Science) using the following keywords: [gut microbiota], [animal microbiome], [gut microbiome 16S] and [captive AND wild AND microbiota]. This search yielded 222 articles on animal microbiomes published between 2014 and 2020. The materials and methods of these articles were analysed to ascertain whether the study met the following criteria: (i) all wild and captive samples were processed using identical procedures, (ii) compared wild and captive animals were phylogenetically closely related (members of the same species or species complex), (iii) captive individuals were born in captivity, or no information was provided about the origin of the captive animals; i.e., wild animals brought into captivity and sampled some time later were excluded, (iv) captive animals that underwent a deliberate selection process (e.g. inbred mice or domestic animals) were also excluded for considering them genetically not comparable to the wild counterparts, and (v) only datasets with sample sizes over 12 individuals were considered for analysis. Raw data were extracted from the databases and repositories indicated in the articles (accession numbers listed in the “Bioinformatic resources”).

### Bioinformatic sequencing data processing

Datafiles from the different studies were (i) stored at the University of Copenhagen’s Electronic Research Data Repository (ERDA), (ii) assigned a unique study identifier and (iii) re-processed in the Danish National Supercomputer for Life Sciences ‘Computerome2’ using a new bioinformatic pipeline we developed for processing data with different characteristics, including sequencing mode, read length and 16S rRNA gene fragment. The entire code can be found in the “Bioinformatic resources''. In short, for each individual dataset, we quality-filtered (mean phred score of q = 25) and (if necessary) trimmed and merged the paired-end reads based on the sequence overlap using AdapterRemoval2^24^. Primers (if present) were trimmed using Cutadapt^25^, and reads were dereplicated with USEARCH Derep^26^ using a relative minimum copy number threshold of 0.01% of the total sequencing depth. Reads were then converted into zero-ratio OTUs using the denoising algorithm UNOISE3^27^, and USEARCH was used to map the reads back to the OTUs and create an OTU table. HS-Blast^28^ was used to assign taxonomy against the non-redundant Silva 132 database^29^, and taxonomic assignments were filtered using different identity thresholds for each taxonomic level: 97% for genus-level taxonomy, 95% for family-level taxonomy, 92% for order-level taxonomy and 90% for higher taxonomic levels^30^. To minimise the impact of incorrectly assigned taxa, taxonomic annotations below these identity thresholds were converted into unclassified, and not considered in downstream analyses. This pipeline yielded relative read abundances assigned to different taxa for each individual dataset analysed.

### Data quality filtering

Individual data files generated by the aforementioned pipeline were aggregated by study and host species into genus-level abundance tables. The two datasets of *Sarcophilus harrisii* retrieved from two different studies were processed independently. Taxonomic resolution was limited to the genus level to maximise taxonomic annotation rate and minimise biases introduced by the different 16S rRNA gene markers employed in the analysed studies. On the one hand, wild animals’ microbial communities often contain taxa that do not map to any catalogued species with enough molecular similarity to assign species-level annotation. On the other hand, the analysed datasets were generated based on the V4, V3–V4 and V1–V3 regions of the 16S rRNA gene (Supplementary Dataset), which hindered comparability at the ASV or zOTU level. We then proceeded to quality-filter the genus-level abundance tables of each species through filtering individuals by minimum sequencing depth, minimum diversity coverage and taxonomic annotation. Only individual datasets with more than 1000 reads and diversity coverage values over 99% were retained, and final genus-level abundance tables that contained at least five animals in each contrasting group were considered for analysis. Since the studied datasets contained traces of dietary items and host DNA, read counts assigned to taxonomic groups not assigned to Bacteria genera, or not present in the LTPs132_SSU release of the SILVA Living Tree (https://www.arb-silva.de/projects/living-tree) used for measuring the phylogenetic relationships among bacteria, were removed to ensure accurate measurements of phylogenetic diversities. In the cases where one group (either wild or captive) outnumbered the other, samples were randomly selected to ensure even sample sizes.

### Diversity and compositional analyses

Diversity and compositional analyses were carried out in the R statistical environment v.3.6.3^31^ and Python 3.8 based on the Hill numbers framework. The operations explained below were conducted using the R packages ape^32^, dendextend^33^, dmetar^34^, hilldiv^35^, meta^36^, metamicrobiomeR^37^, phylosignal^38^, phytools^39^, treedist^40^, vegan^41^, and the python package qdiv^42^. Hereafter functions and their respective packages are displayed as ‘*package::function’*. Statistical significance level was set at a FDR-adjusted p-value of 0.05. All charts and figures in the manuscript were originally generated either in R (full code of all original figures is included in “Bioinformatic resources”) and subsequently modified in Adobe Illustrator to achieve the desired layout without distorting the dimensions of the quantitative elements.

#### Hill numbers

The Hill numbers framework encompasses the group of diversity measures that quantify diversity in units of equivalent numbers of equally abundant taxa^43,44^—in our context bacteria genera. Hill numbers provide a general statistical framework that is sufficiently robust and flexible to address a wide range of scientific questions that molecular ecologists regularly try to answer through measurement, estimation, partitioning and comparison of diversities^45^. To obtain a complete vision of the gut microbiome differences between wild and captive animals, we conducted all our diversity and compositional analyses based on three contrasting Hill numbers based metrics: the so-called dR, which only accounts for richness (i.e., order of diversity 0, whether bacteria taxa were present or not), dRE which considered Richness + Evenness of order of diversity 1 (i.e., the relative abundances of bacteria are proportionally weighed) and dRER, which considered Richness, + Evenness + Regularity (i.e., the phylogenetic relationships among bacteria are accounted for). Detailed explanations of these metrics can be found elsewhere^17,46,47^.

#### Phylogenetic trees

The dRER metric required a Bacterial phylogenetic tree to compute the relatedness among bacterial taxa. As our datasets contained different fragments of the 16S rRNA gene, and thus we were unable to generate a phylogenetic tree directly from our DNA sequence data, we relied on the SILVA Living Tree, and used the LTPs132_SSU release as the reference phylogenetic tree. In addition, the time-calibrated host phylogeny required by the host phylogenetic signal and phylosymbiosis analyses was generated using Timetree^48^.

#### Diversity metrics and meta-analysis

We computed individual-based diversity metrics using the function *hilldiv::hill_div*, and obtained average alpha diversity metrics per species, as well as wild and captive populations per species. We used a Kruskal–Wallis (KW) test as implemented in the function *hilldiv::div_test* to ascertain whether the mean diversity values varied across analysed host species, and a PERMANOVA (PMV) test using *vegan:*:*adonis* function based on the pairwise dissimilarity matrix to test whether host species were compositionally distinct.

Average alpha diversity metrics of wild and captive populations per species were used to conduct a random-effects-model (REM) meta-analysis with raw effect sizes using the function *meta::metacont*. We used the Sidik–Jonkman estimator for the between-study variance and the Knapp–Hartung–Sidik–Jonkman adjustment method. The overall effect was calculated using Hedge's g (SMD) and its 95% confidence interval and p-value. An identical analysis was performed for the entire dataset and two representative subsets of five species, containing only datasets derived from primates and cetartiodactylans. Higgin’s and Thompson’s I^2^ test, Tau-squared T^2^ and Cochran’s *Q* were used for quantifying the heterogeneity between the included species. Due to the high heterogeneity found in the study, we evaluated whether the between-study heterogeneity was caused by outliers with extreme effect sizes, which could be distorting our overall effect. We defined an outlier if the species’s confidence interval did not overlap with the confidence interval of the pooled effect using *dmetar::find.outliers* function.The function detected three outliers in dR metric (GOGO, PEMA and TUTR), four in dRE (GOGO, PEMA, MOCH, EQKI) and seven in dRER (RHBR, PYNE, PEMA, TUTR, MOCH, CENI and AIME). Even when these outliers were excluded from the analysis the I^2^ heterogeneity value was substantial for dR (I^2^ from 79.3 to 70.3%) and moderate for dRE (I^2^ from 80.1 to 60.0%) and dRER (I^2^ from 86.9 to 54.2%) and significant for both (Cochran’s *Q,* p-value < 0.001). We performed a sensitivity analysis removing the outliers from the meta-analysis, yet the results of the random effects model did not change (dR: SMD = 0.345, p-value = 0.075; dRE: SMD = 0.021, p-value = 0.901; dRER: SMD = 0.015, p-value = 0.928). We also performed Graphical Display of Study Heterogeneity (GOSH) plots to explore the patterns of effect sizes and heterogeneity in our data. We used three supervised machine learning (k-means, DBSCAN and the Gaussian Mixture Model) algorithms to detect clusters in the GOSH plot data and determine which studies contribute the most to them automatically using *dmetar::gosh.diagnostics* function.

#### Host phylogenetic signal of wild-captive microbiota differences

We tested whether the diversity and compositional changes were more similar among related host species than species drawn at random through Abouheif's C_mean_ index^49^, as implemented in the function phylosignal::phylosignal. This index was selected for being one of the phylogenetic indices that fulfill most of the criteria for good index and test performance^50^.

#### Wild-captive compositional differences

We first quantified compositional differences between wild and captive animals within each species for the three diversity metrics by means of Jaccard-type overlap-complement derived from the beta diversity values between wild and captive populations using the function *hilldiv::beta_dis.* To test whether the observed differences could be expected by chance or not, we used the Raup-Crick null model extended to the whole continuum of Hill-based regular and phylogenetic dissimilarity indices (^q^RC), as implemented in the *qdiv::null.rcq* function. For dR we used the arguments divType = ‘Jaccard’ and q = 0, for dRE divType = ‘Jaccard’ and q = 1, while for dRER we employed divType = ‘phyl’ and q = 1. Randomisation was performed using the ‘frequency’ procedure, and a significance threshold of 0.05 was established.

#### Classification of compositional differences into predefined scenarios

We used Approximate Bayesian Computation (ABC) to identify which of five predefined scenarios best fit the observed microbiota variation between wild and captive populations in the studied species. Each scenario was characterised as a combination of normal distributions around mean percentages of taxa detected only in wild individuals, shared by both populations and found only in captive individuals. Priors were generated using singular normal distributions and singular value decomposition using a covariance matrix with equal variances of 40%. Scenarios are described in Fig. 2b and details can be found in the “Bioinformatic resources”. Subsequently, we used the function *abc::postpr* to estimate posterior model probabilities and perform model selection based on a multinomial logistic regression as implemented in the function *nnet::multinom*. The scenario with the highest posterior probability was selected as the most representative for each dataset.

#### Compositional similarities among populations within and between host species

We split the abundance table of each species into two subtables only containing either wild or captive individuals, and these subtables were converted into incidence data, which yielded one incidence-based microbiota profile representing each origin (wild and captive) per dataset. Pairwise dissimilarities of all dataset*origin combinations were computed using *hilldiv::pair_dis*. For each of these 50 datasets, we identified the closest match and calculated the percentage of cases in which the closest match was not the wild or captive counterpart of each species.

#### Taxonomic enrichment analysis and meta-analysis

We analysed taxonomic relative abundance differences between wild and captive populations per host species using Generalized Additive Models for Location, Scale and Shape (GAMLSS) as implemented in *metamicrobiomeR::taxa.compare*. Subsequently, random effects meta-analysis models were applied, using *metamicrobiomeR::meta.taxa*, to the pool of estimates and their standard errors to evaluate the overall effects and heterogeneity across the host species. The minimum prevalence for considering microbial taxa in the meta-analysis was set in 33% of the analysed datasets.

#### Correlation and topological differences between wild- and captive-data derived host comparisons

We computed two sets of pairwise dissimilarities of the microbiota profiles among species, by separately considering only wild and captive individuals. The correlation between the two dissimilarity matrices was computed in terms of Pearson Product-Moment Correlation test using the function *stats::cor.test*. For each pairwise comparison, we also extracted the evolutionary distance in terms of millions of years of divergence time from the host species phylogeny using *ape::cophenetic.phylo* function. Pairwise dissimilarity matrices derived from wild and captive individuals were subsequently used to generate compositional cladograms based on hierarchical clustering (UPGMA method). These trees were contrasted with the host phylogenetic tree to compute phylosymbiosis in terms of generalised Robinson–Foulds distances, as implemented in the *TreeDist::JaccardRobinsonFoulds* function. To test whether wild and captive datasets yield different phylosymbiosis effect sizes, we performed a t-test on phylosymbiosis metrics iteratively computed from wild and captive data of 100 randomly selected subsets of ten host species.

## Supplementary Information

Supplementary Information.

## Data Availability

All raw DNA sequences are available in NCBI SRA. Bioinformatic procedures and relevant metadata are included in the Supplementary Information. The studies included in the meta-analysis are listed in the Supplementary Dataset. Bioinformatic resources, including accession numbers, scripts, data files and raw figures have been archived in Zenodo under the 10.5281/zenodo.5594740. Bioinformatic Code A (613 lines) contains the pipeline used for the re-generation of gut microbiota profiles from raw DNA sequence data. Bioinformatic Code B (1695 lines) contains the pipeline employed for the diversity analyses.
